# An Infant Monstrosity

**Published:** 1871-10

**Authors:** 


### An Infant Monstrosity.

The lecture-room of the Jefferson Medical College was filled at noon, the other day, to witness the clinical examination of a wonder in physiology, the two-headed baby. There was present, by invitation, a number of distinguished gentlemen, physicians, public men, and scientists, and the usual number of fledgling doctors. Among the former were Profs. Gross, Pancoast and Rand, of the Jefferson; Drs. Gross, Maury and Pancoast; Edward Shippen and William V. McKean, Esqs., and others.

Shortly after 12 o'clock, Dr. Getchell, the clinical lecturer upon diseases of women and children, made his appearance, and the infant being brought in and placed upon the table, the Doctor proceeded to enter into the history of monsters in general, and of this little wonder in particular. He stated that Dr. Gross had been summoned to see the infant at the Museum, and had called him in to consult with him. Together they had come to the conclusion that the infant was an object of such interest as to merit the attention of the medical public. Through the kindness of Mr. Dixey they had been enabled to present it at the present lecture.

The child was born in Morrow county, Ohio, upon the 12th of October last, and is now accordingly about seven months old. Its delivery was easy and natural, its birth being accomplished before medical attendance was called. There was one cord and one placenta for the new comer. It weighed at its birth twelve pounds, and was in all respects a hearty, healthy child. Since its birth the child has enjoyed good health, suffering only from the slight and natural ailments of early infancy. One child was larger than the other, but both are in a healthy condition at the present time.

The child has two heads, both of which are vivacious and even pretty. It has one continuous spine, with what might be called three legs, and one composed of two legs united together, with one compound foot, and eight toes. In other respects it resembles other children. The Doctor then entered into the history of other monsters, and showed a number of portraits from antique and cotemporaneous works of creatures of the same conformation. In conclusion, he said that on the whole this infant must be regarded as unique, as it had lived longer than any of its predecessors in monstrosity, and still gave promise of long life to come.—*Phila. Press.*

There is no exaggeration in the above description, as we are assured by Mr. Dixey, who has the child on exhibition at the American Museum, in Philadelphia.

KEEP your rooms cool in hot weather by hanging up a wet cloth (the larger the better), securing the best ventilation possible. A reduction of twelve degrees can thus be secured without inconvenience.

A COLLOSSAL bronze statute of Harvey, discoverer of the circulation of the blood, is to be erected in Central Park, New York. Verily, honor to whom honor is due.

THE Gas-light Journal urges the necessity of legal enactment preventing the sale of gasoline and burning fluids for ordinary uses. An eye single to business.

"ASTONISHING cure for consumption," as the old lady said when she sprinkled snuff on her boarders' hash.

Better disburse money in improving a home than on thousands of things on which we too often well nigh throw it away.—*Fowler.*

Attention is called to our Premiums offered to those who desire to solicit subscriptions to the BISTOURY. See cover.

